# The Membrane-Targeting Synergistic Antifungal Effects of Walnut-Derived Peptide and Salicylic Acid on Prickly Pear Spoilage Fungus

**DOI:** 10.3390/foods14060951

**Published:** 2025-03-11

**Authors:** Yue Hu, Na Liu, Caiqing Ma, Difeng Ren, Dujun Wang, Yueling Shang, Fengwei Li, Yongmei Lyu, Chen Cai, Long Chen, Wenjing Liu, Xiaohong Yu

**Affiliations:** 1 School of Marine and Bioengineering, Yancheng Institute of Technology, Yancheng 224051, China; huyue@ycit.edu.cn (Y.H.); 15895515790@163.com (N.L.); 13862214503@163.com (C.M.); wangdj@ycit.edu.cn (D.W.); ylshang33@126.com (Y.S.); lifengwei1980@126.com (F.L.); lyu.yongmei@ycit.edu.cn (Y.L.); 15052263031@163.com (C.C.); 15861040779@163.com (L.C.); 19551506879@163.com (W.L.); 2 State Key Laboratory of Efficient Production of Forest Resources, Beijing Key Laboratory of Food Processing and Safety in Forestry, Department of Food Science and Engineering, College of Biological Sciences and Biotechnology, Beijing Forestry University, Beijing 100083, China; rendifeng@bjfu.edu.cn

**Keywords:** peptide, salicylic acid, synergistic antifungal, cell membrane interactions, cell component release

## Abstract

Fermented walnut (FW) meal exhibits antifungal activity against *Penicillium victoriae* (the fungus responsible for prickly pear spoilage), which is mainly attributed to the synergistic effect of antimicrobial peptides and salicylic acid (SA). This study aimed to investigate the synergistic mechanism between YVVPW (YW-5, the peptide with the highest antifungal activity) and SA against the cell membrane of *P. victoriae*. Treatment enhanced prickly pear’s rot rate, polyphenol concentration, and superoxide dismutase (SOD) activity by 38.11%, 8.11%, and 48.53%, respectively, while reducing the microbial count by 19.17%. Structural analyses revealed β-sheets as YW-5′s predominant structure (41.18%), which increased to 49.0% during SA interaction. Molecular docking demonstrated YW-5′s stronger binding to β-(1,3)-glucan synthase and membrane protein amino acids via hydrogen bonds, hydrophobic forces, and π-π conjugate interactions. Spectroscopic analyses demonstrated SA’s major role in YW-5 synergy at the interface and polar head region of phospholipids, enhancing lipid chain disorder and the leakage of cell components. Malondialdehyde and SOD levels increased nearly two-fold and six-fold when treated with YW-5/SA, and YW-5 showed a more pronounced effect. Scanning electron and transmission electron microscopy confirmed that SA caused greater damage to spore morphology and cell ultrastructure. These findings support this formulation’s functions as an efficient antifungal substance in fruit storage.

## 1. Introduction

Prickly pear (*Rosa roxburghii* Tratt) has been used in Chinese Miao medicine for the treatment of digestive disorders and diarrhea for 324 years [1]. It is rich in vitamin C (2000–3000 mg 100 g^−1^ FW), superoxide dismutase (SOD, 20-fold to 50-fold more than grapes), and flavonoids (20-fold more than kiwi) and has excellent antioxidant and anti-inflammatory activity [2]. Prickly pear ripens in hot and humid conditions, leading to fungal contamination, mycotoxin production, and significant postharvest losses [3]. While antimicrobials can reduce postharvest losses of agricultural products, registered chemically synthesized antimicrobials such as imazalil and prochloraz exhibit limitations, including low residue levels, unclear mechanisms of action, and the development of drug resistance [4].

Antimicrobial peptides (AMPs) are marked by a combination of cationic and hydrophobic amino acids. These peptides demonstrate broad-spectrum antimicrobial activity, low toxicity, and resistance to drug tolerance, and their efficacies are markedly influenced by their charge, hydrophobicity, and secondary structure [5,6]. The antimicrobial capabilities of protein-abundant foods primarily stem from AMPs produced by different proteases during fermentation [7]. AMPs inevitably interact with phenolic compounds through alterations in their secondary structure and characteristics during fermentation [8]. Generally, phenolic molecules of higher molecular weight exhibit stronger binding interactions with phenolic compounds [9]. Phenolic compounds can have either synergistic effects on peptides or negative effects by masking the peptide’s active site [10].

Walnut (*Juglans regia* L.) meal is rich in protein (40–45%) and includes all eight essential amino acids. Additionally, the meal exhibits a polyphenol concentration of 2.92% with twelve phenolic compounds [11]. This consumer by-product holds significant value and requires urgent utilization. Currently, the majority of walnut meal is still utilized for animal feed or discarded, leading to resource waste and environmental pollution. In a previous study, fermented walnut meal (FW) exhibited antifungal activity against *Penicillium victoriae*, the dominant fungus of *Penicillium* responsible for prickly pear spoilage. Ultrasonic-assisted antifungal film loaded with FW not only accelerated reducing the relative abundance of *Penicillium* but also decreased the fungal count and diversity of prickly pear during storage [12]. YVVPW (YW-5) and SA obtained from FW was found to be a potent synergistic combination against *P. victoriae*. Three hydrogen bonds formed via -OH, C=O, N-H, and C-H, and π-π stacking occurred via the benzene ring of SA and the five-membered ring of the tryptophan residue [13]. However, the detailed antifungal mechanisms underlying this synergistic combination have not yet been investigated.

In this study, the synergistic antifungal mechanism of YW-5 and SA (YW-5/SA) derived from FW on the cell membrane of *P. victoriae* was explored. The preservation effect of FW on prickly pear was evaluated. The structural characteristics of YW-5 were analyzed using Fourier-transform infrared (FTIR) and circular dichroism (CD) spectroscopy, and the interaction forces and binding sites between YW-5/SA and the cell membrane were determined using molecular docking, FTIR, and Raman spectroscopy. Additionally, the release of cell components, membrane lipid peroxidation, and the morphology and ultrastructure of *P. victoriae* spores were also assessed. These findings provide insights into the synergistic effects of antimicrobials, offering an alternative for fruit preservation.

## 2. Materials and Methods

### 2.1. Materials and Reagents

Prickly pears were harvested in Guizhou Province and shipped to Jiangsu within 2 days after pre-cooling. The intact prickly pears were placed in cold storage at 4.0 ± 1.0 °C with a relative humidity of 90 ± 5%. FW preparation followed the methodology described in our earlier research [13], and the polyphenol and peptide contents were 74.25 ± 0.09 mg/100 mL and 15.27 ± 0.15 mg/mL, respectively. LB nutrient and potato dextrose agar (PDA) were obtained from Beijing AoBoxing Bio-Tech Co., Ltd. (Beijing, China). SA and gallic acid were procured from Yuanye Biotechnology Co., Ltd. (Shanghai, China). YW-5 (purity ≥98%) was produced via Fmoc solid-phase methodology utilizing a peptide synthesizer (Applied Biosystems, Warrington, UK). Tris(hydroxymethyl) aminomethane, trifluoroethanol (TFE), carboxymethyl chitosan, and gelatin (source: bovine bone, bloom 260) were supplied by Macklin Biochemical Co., Ltd. (Shanghai, China). The polyethersulfone (PES) needle filter was purchased from Jinteng experimental equipment Co., LTD (Tianjin, China). HCl, chloroform, and methanol were ACS reagent grade and procured from Beijing Chemical Works (Beijing, China).

### 2.2. Preservation Effect of FW on Prickly Pear

The antifungal film loaded with FW was prepared according to the method outlined in a previous study [12]. Briefly, GL (2%, *w*/*v*) and CMCS (2%, *w*/*v*) were mixed at a ratio of 1:2, and 1.0 wt% of glycerol was added as a plasticizer. The obtained film-forming solution was immediately ultrasonically treated with 560 W for 5 min using an ultrasonic processor (Biosafer 650–92, Thermo Fisher, Waltham, MA, USA) and stirred for 55 min to obtain ultrasonic-assisted CMCS/GL (U-CMCS/GL) film-forming solution. Then, 1% FW was added to the blend solution and stirred for 30 min to prepare the final U-CMCS/GL/FW film-forming solution. The randomly selected prickly pears were divided into three groups, including untreated, U-CMCS/GL, and U-CMCS/GL/FW groups, and each group contained 150 prickly pears. The prickly pears were soaked for 1 min in film solution and then surface-dried in a sterile airflow for 2 h, with the untreated group as the control. The three groups were placed in Petri dishes (plastic film sealed and punctured 10 times for aerobic circulation). The samples were stored at near-freezing temperature (−0.8 to −0.4 °C) [12], and measured at 0, 10, 20, 30, 40, and 50 days. All experiments were performed in triplicate.

The rot rate was calculated as the ratio of the number of rotted prickly pears to the total number. Titratable acidity was determined according to Chinese GB 12293-90 [14]. Prickly pear juice was diluted with distilled water and then titrated with 0.10 M sodium hydroxide (grams of malic acid equivalents per gram of fruit). A hand-held refractometer (PAL-1, Atago, Tokyo, Japan) was used to measure the total soluble solids of the prickly pear during storage. SOD activity was assessed using a SOD assay kit from Nanjing Jiancheng Bioengineering Institute (Nanjing, China). Total polyphenol content (mg of gallic acid equivalents per gram of prickly pear) was ascertained by utilizing the Folin–Ciocalteau method, based on a calibration curve constructed with reagent-grade gallic acid over the relevant concentration range [15]. Aerobic plate count was determined following Chinese GB 4789.2-2016 [16]. Next, 25 g of prickly pear was homogenized in 225 mL of 0.9% (*w*/*v*) sterile saline water for 1 min at 8000 rpm. Then, a 10-fold serial dilution was performed with sterile saline water, and 1 mL of each sample was spread-plated onto LB nutrient agar for incubation at 37 °C for 24 h to determine the aerobic plate count.

### 2.3. Structural Characterization of YW-5

#### 2.3.1. FTIR Analysis of YW-5 

The FTIR analysis of YW-5 in its solid state was conducted by utilizing a spectrometer in ATR mode (Nicolet 6700, Thermo Fisher Scientific). The spectrum was recorded in transmission mode (400 to 4000 cm^−1^), comprising 32 scans at a precision of ±4 cm^−1^. The experimental outcomes were evaluated by utilizing OMNIC 8.2 software and applying the GaussAmp function in PeakFit software v. 4.12 for fitting. The relative content of each secondary structure was computed by integrating the area under each sub-peak and correlating it with the respective secondary structures.

#### 2.3.2. CD Spectrum

To analyze the secondary structure of YW-5 in liquid conditions, CD spectroscopy (J-1500, JASCO, Tokyo, Japan) was employed by utilizing a Peltier component for temperature control at 25 °C. The CD measurements were obtained within 195–250 nm by employing a quartz cuvette featuring a 1 mm diameter, 2 nm slit width, and 60 nm/min scanning velocity. YW-5 (0.2 mg/mL) underwent examination in multiple solutions: 10 mM HCl for gastric acid stimulation after feeding; 10 mM sodium phosphate buffer (PBS, pH 7.2) to represent aqueous conditions; 50% TFE to reflect membrane hydrophobicity; and 0.2 mg/mL SA to examine YW-5 and SA interactions. The secondary structural elements of YW-5 were calculated using the DichroWeb database (http://dichroweb.cryst.bbk.ac.uk/html/home.shtml, accessed on 10 March 2025), while the CONTIN algorithm facilitated the examination of variations based on CD spectra fitting peak positions.

### 2.4. Theoretical Calculation of Interaction Forces

#### 2.4.1. Molecular Docking with Rho1 GTPase

SA and Rho1 GTPase underwent hydrogenation, structural refinement, and energy optimization through the utilization of Schrodinger Maestro 11.9 platform [17]. The docking analysis was executed through the Glide module. Rho1 GTPase underwent minimization as the receptor by employing standard parameters of the LigPrep module before docking implementation. A grid container was established with 10 × 10 × 10 Å measurements to encompass Rho1 GTPase. The binding forces were evaluated between Rho1 GTPase and SA.

#### 2.4.2. Molecular Docking with Amino Acids from Membrane Protein

For the docking experiments, YW-5 and SA were used as ligands; Tryptophan (Trp), Phenylalanine (Phe), and Tyrosine (Tyr) served as receptors. The grid box was centered on the centroid of the receptor and set to a size 40 × 40 × 40 Å. Molecular docking was executed by utilizing AutoDock, and the binding effects were visualized with PyMOL 2.1 software. The Lamarckian genetic algorithm was utilized for the calculations (population size = 150, maximum energy evaluations = 25 million, maximum generations = 2000, crossover rate = 0.8, mutation rate = 0.02, and 10 independent docking runs). The resultant docking conformations were assessed per their binding free energy values.

### 2.5. Spectroscopic Analysis of the Interaction Site at the Cell Membrane

#### 2.5.1. Mimic *P. victoriae* Membrane Liposome Preparation

The mimic *P. victoriae* membrane liposome was prepared per the procedure established by Tang et al. [18], with minor modifications. A spore suspension of *P. victoriae* (10^5^ CFU/mL) was dispersed in potato dextrose water medium and cultured for 48 h at 28 °C while being shaken at 120 rpm. The resulting sediment was collected through centrifugation at 5000 rpm for 7 min at 4 °C and subsequently resuspended in Tris-HCl buffer (25 mmol/L, pH 7.5). The cell suspension was combined with a chloroform–methanol solution (1:2, *v*/*v*) and agitated for 90 min. After stirring, an equal volume of distilled water–chloroform (1:1, *v*/*v*) was added and stirring was continued for 30 min. The chloroform layer was isolated, and the aqueous phase underwent two chloroform extractions. The chloroform fractions were combined, and the solvent was eliminated via rotary evaporation to yield the lipid extract.

Next, the *P. victoriae* lipid extract underwent dissolution in chloroform–methanol (2:1, *v*/*v*) and was concentrated into a thin layer via rotary evaporation under decreased pressure. The residual solvent was eliminated through high vacuum exposure overnight, followed by film hydration with the utilization of Tris-HCl buffer (10 mM, pH 7.2). The lipid membrane suspension was subjected to ultrasonication at 20 kHz, 300 W, and 3:3 (s/s) for 20 min. The resulting mimic membrane liposome suspension was passed through a 0.45 μm PES needle filter and underwent lyophilization.

#### 2.5.2. FTIR and Roman Interaction Site Analysis

The minimal inhibitory concentrations (MICs) of YW-5, SA, and YW-5/SA were ascertained to be 16 mg/mL, 4.4 mg/mL, and 2.0 mg/mL for YW-5 and 0.55 mg/mL for SA. The membrane liposome was resolved in Tris-HCl buffer (10 mM, pH 7.2). During this stage, 1 MIC and 2 MIC of YW-5, SA, and YW-5/SA were added to the liposome suspension for 4 h (1:5, *v*/*v*). The PBS buffer-treated liposome suspension was used as the blank control. The treated mimic membrane liposome was lyophilized and analyzed using FTIR and Raman spectroscopy.

For FTIR, a spectrometer was used in ATR mode (Nicolet 6700, Thermo Fisher Scientific). The spectrum was collected in transmission mode over the range of 400 to 4000 cm^−1^ with 32 scans and a resolution of ±4 cm^−1^. The outcomes were collected by utilizing OMNIC software 8.2. The Raman spectrum of the mimic membrane liposome was measured using a Raman microscope (LabRAM HR Evolution, Horiba, Paris, France). The excitation wavelength was set to 785 nm and scanned in the range of 1000–1200 cm^−1^.

### 2.6. Release of Cell Components

#### 2.6.1. FTIR Analysis of Cell Components

A spore suspension of *P. victoriae* (1 × 10^5^ CFU/mL) was established in potato dextrose water medium and cultivated for 48 h at 28 °C under continuous agitation at 120 rpm. The sediment was collected through centrifugation at 8000 rpm for 5 min at 28 °C and subsequently resuspended in PBS. SA and YW-5 were introduced to the cultures at a final concentration of 1 MIC, along with various combined concentrations of YW-5 and SA (2 + 0.55 mg/mL, 2 + 1.1 mg/mL, 4 + 0.55 mg/mL, 4 + 1.1 mg/mL, 4 + 2.2 mg/mL, and 8 + 1.1 mg/mL, 8 + 2.2 mg/mL). The cultures underwent incubation at 28 °C. Absolute ethyl alcohol served as a negative control. Following an 8 h incubation period, the cells were gathered, centrifuged at 8000× *g* for 2 min, and cleansed three times with PBS. The mycelium was lyophilized, and the changes in cell components were analyzed via FTIR using the method outlined in Section 2.5.2.

#### 2.6.2. Intracellular Pyruvic Acid, Protein, and OD260

The procedure followed the approach outlined by Ju et al. [19], with minor adjustments. The mycelium was grounded with liquid nitrogen, and the slurry was centrifuged at 2000 rpm for 15 min at 4 °C. The resulting supernatant was utilized to ascertain intracellular pyruvic acid, protein content, and OD_260_. The effects of 1 MIC SA, YW-5, YW-5/SA, and 2 MIC YW-5/SA treatments on intracellular protein and pyruvic acid levels were detected by utilizing the total protein assay kit and pyruvate assay kit from Nanjing Jiancheng Bioengineering Institute (Nanjing, China). OD_260_ was determined by using a UV spectrophotometer at 260 nm.

### 2.7. Malondialdehyde Content, Superoxide Dismutase Activity, and Lipase Activity Assay

A spore suspension of *P. victoriae* (1 × 10^5^ CFU/mL) was established in potato dextrose water medium and cultivated for 48 h at 28 °C under continuous agitation at 120 rpm. The sediment was collected through centrifugation at 8000 rpm for 5 min at 28 °C and subsequently resuspended in PBS. SA and YW-5 were introduced to the cultures at a final concentration of 1 MIC. The cultures underwent incubation at 28 °C. Absolute ethyl alcohol served as a negative control. Following an 8 h incubation period, the cells were gathered, centrifuged at 8000× *g* for 2 min, and cleansed three times with PBS. The mycelium was ground with liquid nitrogen and a slurry was prepared by adding PBS buffer. Then, the slurry was centrifuged at 2000 rpm for 15 min at 4 °C. The resulting supernatant was utilized to ascertain MDA content, SOD activity, and lipase activity using MDA, SOD, and lipase assay kits from Nanjing Jiancheng Bioengineering Institute (Nanjing, China).

### 2.8. Scanning Electron Microscopy (SEM) and Transmission Electron Microscopy (TEM)

The fungal mycelium (Section 2.7) underwent vacuum incubation for 10 min in 2.5% glutaraldehyde mixed with 0.1 M phosphate buffer (pH 7.0). Following this, the specimens were washed in identical buffer solution for 8 h at 4 °C, followed by sequential dehydration in ascending ethanol concentrations (50%, 70%, and 90%), CO_2_-based critical-point drying, and placement on specimen stubs. Gold coating was applied to the specimens before examination with a SEM (SU8010, Hitachi, Tokyo, Japan). Anhydrous ethanol-treated mycelium was regarded as a negative control.

For TEM analysis, the specimens underwent sequential dehydration by utilizing ascending concentrations of ethanol (50%, 70%, and 90%) with 15 min intervals. Subsequently, the specimens were processed with acetone embedding mixture (3:1) for 2–3 h followed by acetone embedding mixture (1:1) for 2–3 h. Following immersion in pure embedding mixture overnight, the specimens underwent polymerization in a temperature-controlled oven (37 °C overnight, 45 °C for 12 h, and 60 °C for 24 h). Ultrathin slices were prepared (approximately 50–60 nm thickness), subjected to staining with 3% uranyl acetate and lead citrate, and observed under a TEM (H-7650, Hitachi, Japan).

### 2.9. Statistical Analysis

Each experiment was performed three times, and the experimental findings are presented as the mean ± standard deviation. The outcomes were evaluated by utilizing Excel 2016 and GraphPad Prism 8.3. Differences between means were assessed via one-way analysis of variance (ANOVA, *p* < 0.05) by employing SPSS software (Version 26.0, IBM SPSS Inc., Armonk, NY, USA).

## 3. Results and Discussion

### 3.1. Enhancement of Freshness Capacity

**Figure 1:**
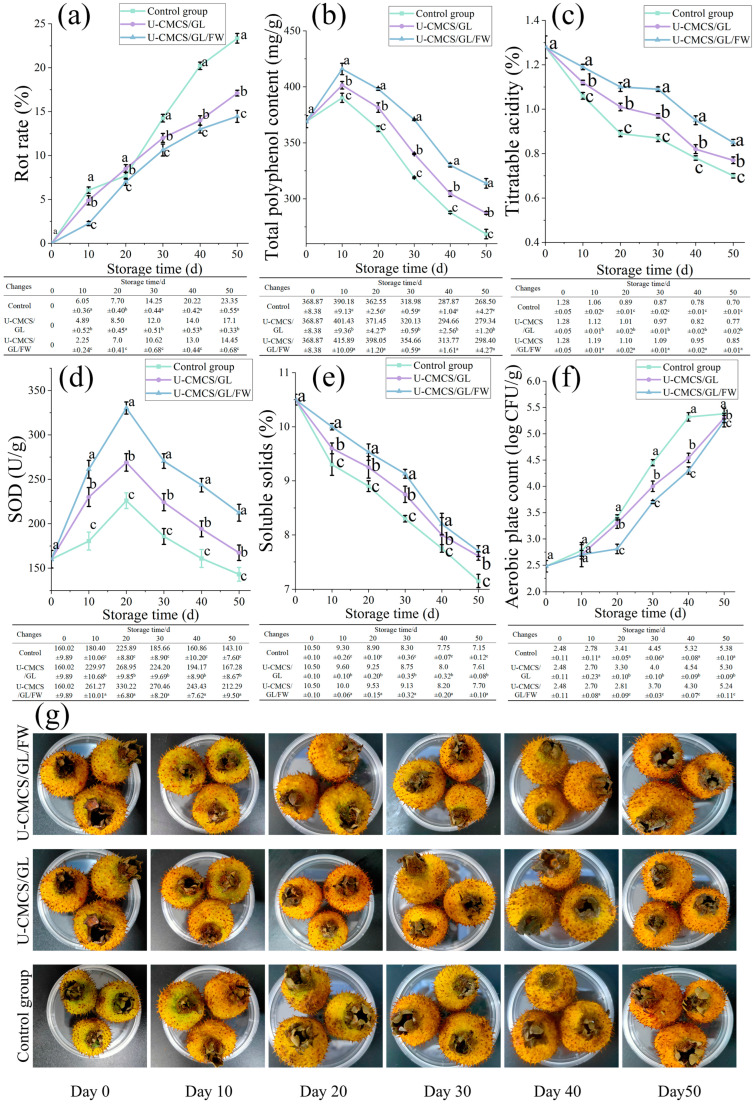
Rot rate (**a**), total polyphenol content (**b**), titratable acidity (**c**), SOD activity (**d**), soluble solids (**e**), aerobic plate count (**f**), and appearance (**g**) of preserved prickly pears during storage. Different lowercase letters indicate significant differences (*p* < 0.05) of freshness capacity among samples.

The color of the prickly pear changed from yellow–green to dark yellow during storage (Figure 1). After 30 days, the control group exhibited browning, speckling, and partial rot, while the coated group remained firmer with fewer speckles. The rot rate is a key factor limiting the post-harvest shelf life of fruits as it directly indicates the degree of spoilage [20]. On the 20th day of storage, the rot rate in the U-CMCS/GL group reached 8.0 ± 0.45%, the highest observed. However, by the end of storage, the rot rate in the U-CMCS/GL/FW group was 38.11% lower than that of the control group. The main cause of decreased titratable acidity and soluble solids in prickly pear is the consumption of organic acids and sugars. Notably, the soluble solids in all three groups decreased rapidly by the 10th day. By the end of storage, the soluble solids in the control, U-CMCS/GL, and U-CMCS/GL/FW groups decreased by 31.90%, 27.52%, and 26.67%, respectively. Similarly, titratable acidity decreased by 45.31%, 39.84%, and 33.60%, respectively. Phenolic compounds are among the primary antioxidants in prickly pear and contribute to its astringent taste [21]. During the early stages of storage, the total polyphenol content in all three groups increased gradually, likely due to increased phenylalanine ammonia–lyase activity as the tissue aged that catalyzed the synthesis of phenolic compounds from Phe [22]. At this stage, the total polyphenol content in the control, U-CMCS/GL, and U-CMCS/GL/FW groups was 390.18 ± 4.13 mg/g, 401.43 ± 3.36 mg/g, and 415.89 ± 5.09 mg/g, respectively. However, the polyphenol content in all groups later declined, likely due to oxidation into quinones. The U-CMCS/GL/FW group exhibited the smallest decrease in polyphenol content (19.10% reduction by the end of storage). SOD activity in the control, U-CMCS/GL, and U-CMCS/GL/FW groups peaked on the 20th day of storage, with values of 225.89 ± 8.80 U/g, 268.95 ± 9.85 U/g, and 330.22 ± 6.80 U/g, respectively. Afterward, SOD activity declined to its lowest point by the end of storage. The U-CMCS/GL/FW group maintained the highest SOD activity at 212.29 ± 9.5 U/g, likely due to the stronger oxygen barrier properties of the coating and the inclusion of antioxidant-rich FW. In this study, 5.0 log CFU/g was considered as the microbial safety threshold. Aerobic counts increased gradually across all three groups during storage. By the 40th day, the aerobic plate count in the control group reached 5.32 ± 0.08 log CFU/g, whereas in the U-CMCS/GL (4.54 ± 0.09 log CFU/g) and U-CMCS/GL/FW (4.30 ± 0.07 log CFU/g) groups, the counts remained below 5.0 log CFU/g. By the 50th day, all three groups had exceeded the 5.0 log CFU/g threshold. These findings suggest that FW could not only slow nutrient degradation but also provide protection against microbial infections in prickly pear.

### 3.2. Structural Characterization

**Figure 2:**
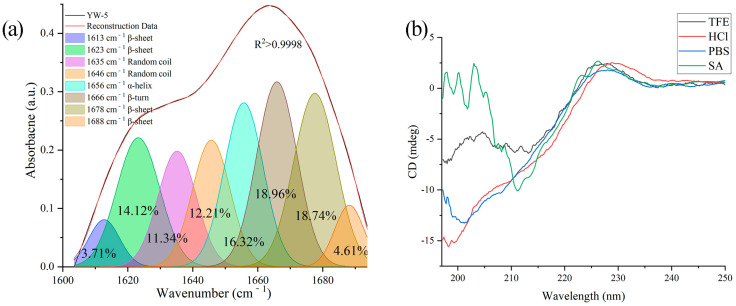
Secondary structure spectra of YW-5 as determined using FTIR (**a**) and CD (**b**).

AMPs are influenced by hydrophobic interactions, hydrogen bonding, and Van der Waals forces in different solutions [23]. In this study, FTIR was used to determine the secondary structure of YW-5 in its solid state (Figure 2a). The 1600–1700 cm⁻^1^ range was focused on fitting the protein secondary structure. The characteristic wave numbers for α-helical, β-sheet, β-turn, and random coil motifs were 1615–1660 cm⁻^1^, 1600–1640 cm⁻^1^, 1661–1700 cm⁻^1^, and 1641–1650 cm⁻^1^, respectively [24]. In its solid state, YW-5 exhibited 41.18% β-sheets, 23.54% random coils, 18.96% β-turns, and 16.32% α-helical structures (Table 1).

The secondary structures of YW-5 were determined in four different solutions using CD analysis (Figure 2b). In all four solutions, YW-5 exhibited α-helix, β-sheet, β-turn, and random coil structures. The β-sheet remained the predominant secondary structure (49.0%) in the presence of SA. However, there were no significant differences in the β-sheet and random coil content of YW-5 across acidic, aqueous, and hydrophobic environments. In these conditions, random coils were the most abundant (41–46%), followed by β-sheets (32–39%), indicating that the secondary structure of YW-5 changes when transitioning from a solid to a liquid state (Table 1). In contrast, Wang et al. [25] found that the MCNDCGA peptide from *Moringa oleifera* seeds exhibited antibacterial activity against *Staphylococcus aureus*, with β-turns and α-helices being the dominant secondary structures in the solid state and β-sheets and random coils being dominant in acidic, aqueous, and hydrophobic environments. Based on this, YW-5 might primarily interact with fungal cell membranes through its random coil structure, while its β-sheet structure facilitates interactions with SA.

### 3.3. Interaction Forces Determined via Molecular Docking

**Figure 3:**
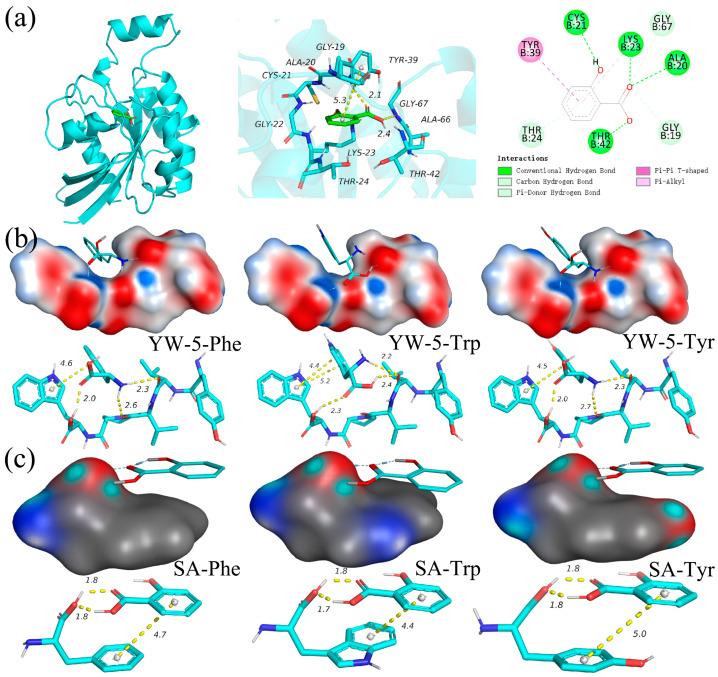
Antifungal and synergistic mechanisms as investigated via molecular docking. The 3D structures and conformational interactions between SA and Rho1 GTPase (**a**), between YW-5 and amino acids (**b**), and between SA and amino acids (**c**). Yellow horizontal lines indicate hydrogen bond distance or π-π stacking. Red, blue, dark blue, and gray lines indicate O, C, N, and H, respectively.

β-1,3-glucan synthase (GS, EC 2.4.1.34) is responsible for synthesizing the fungal cell wall polymer β-(1,3)-glucan, and Rho1 GTPase serves a crucial function in fungal cell wall biosynthesis [26]. Figure 3a shows the conformational interactions between SA and Rho1 GTPase. Our previous study demonstrated that YW-5 formed hydrogen bonds with Lys-123, Lys-167, Pro-36, and Val-38, and π-π conjugated interactions occured between the benzene ring and Tyr-39, Cys-25, and Phe-35 [13]. Similarly to YW-5, hydrogen bonding, hydrophobic interactions, and π-π conjugated interactions were the primary forces between SA and Rho1 GTPase. The binding affinity of SA to Rho1 GTPase (−6.65) was lower than that of YW-5 (−8.5). SA formed hydrogen bonds with Cys-21, Lys-23, Ala-20, Thr-42, and Tyr-167, and π-π conjugated interactions occurred between the benzene ring and Tyr-39.

Molecular docking was also used to analyze the noncovalent interactions between YW-5 and the key amino acids of the cell membrane, as well as between SA and these amino acids, including Trp, Tyr, and Phe [19,27]. The primary interaction forces between YW-5 and SA with these amino acids were hydrogen bonding, hydrophobic interactions, and π-π conjugated interactions (Figure 3b,c). YW-5 exhibited stronger binding affinity compared to SA. The binding affinities for YW-5 with Trp, Tyr, and Phe were −5.92 kcal/mol, −5.47 kcal/mol, and −5.35 kcal/mol, respectively. In comparison, the binding affinities for SA with Trp, Tyr, and Phe were −5.13 kcal/mol, −4.53 kcal/mol, and −4.79 kcal/mol, respectively. YW-5 formed three hydrogen bonds with Trp at distances of 2.3 Å, 2.4 Å, and 2.2 Å, while SA formed two strong hydrogen bonds at distances of 1.7 Å and 1.8 Å, markedly shorter than the typical hydrogen bond distance (3.5 Å). Additionally, benzene rings of both YW-5 and SA formed strong π-π conjugated interactions, with the benzene rings of Trp, Phe, and Tyr differing by only a single phenolic hydroxyl group. YW-5 had a slightly higher affinity for Tyr, whereas SA had a marginally better affinity for Phe. Overall, YW-5 demonstrated a stronger noncovalent interaction with the cell wall and membrane compared to SA.

### 3.4. The Impact of YW-5 and SA on Cell Membrane Interactions

**Figure 4:**
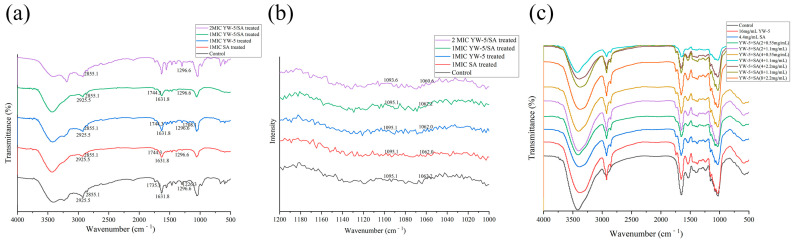
Synergistic effects of YW-5 and SA on the interaction site of cell membranes as analyzed using FTIR (**a**) and Raman (**b**) spectroscopy. Synergistic effects of YW-5 and SA on the release of cell components as revealed using FTIR (**c**).

Figure 4a presents the FTIR spectra of mimic *P. victoriae* membrane liposomes treated with 1 MIC YW-5, SA, YW-5/A, and 2 MIC YW-5/A. After treatment with 1 MIC YW-5, SA, YW-5/SA, and 2 MIC YW-5/SA, the characteristic peak at 1731.3 cm⁻^1^ shifted to 1744.3 cm⁻^1^. The relative intensity of the peak for SA-treated mimic membrane liposomes was markedly lower compared to YW-5-treated liposomes, indicating that YW-5 and SA interacted synergistically with the interface region of the phospholipids, with the synergistic effect being primarily attributed to SA [28]. There was no change in the absorption peaks at 2925.5 cm⁻^1^ and 2855.1 cm⁻^1^, suggesting that YW-5/SA did not affect the fatty acyl chains of the phospholipids. Interestingly, the absorption peak at 1226.3 cm⁻^1^ for mimic membrane liposomes treated with YW-5 shifted to 1208.1 cm⁻^1^ but disappeared after treatment with 1 MIC SA, YW-5/SA, and 2 MIC YW-5/SA. This shift indicates that YW-5 and SA synergistically interacted with the head region of the phospholipids, with SA being the primary contributor to this interaction [21]. Overall, the FTIR results suggest that YW-5/SA interacted with the mimic membrane liposomes primarily at the interface and polar head regions of the phospholipids.

The peaks of the Raman spectrum of mimic *P. victoriae* membrane liposomes at 1062 cm⁻^1^ and 1096 cm⁻^1^ correspond to the trans-side conformation of the fatty acid carbon chain, primarily caused by C-C stretching vibrations (Figure 4b). The peak at 1062 cm⁻^1^ is attributed to the C-C skeleton twisted conformation, reflecting the B_1_g mode of vibration of carbon chain fragments in all-trans fatty acids. The peak at 1096 cm⁻^1^ is mainly due to C-C skeleton vibrations (PO_2_⁻ stretching vibrations), indicating the vibration mode of the lipid chain structure with a side conformation. The ratio of the peak intensities I_1096_/I_1062_ represents the ratio of the para-dipoid conformation to the trans conformation and is used to characterize the disorder degree of the lipid chain [29]. Compared to the untreated mimic *P. victoriae* membrane liposomes, the I_1096_/I_1062_ peak strength ratio increased by 3.13%, 1.04%, 10.42%, and 17.71% following treatment with 1 MIC SA, YW-5, YW-5/SA, and 2 MIC YW-5/SA, respectively. These results indicate that YW-5/SA treatment enhanced the fluidity and lipid chain disorder of the *P. victoriae* cell membrane, with SA serving a predominant function.

### 3.5. The Impact of YW-5 and SA on Cell Component Release

Figure 4c shows the FTIR results depicting changes in the cell components of *P. victoriae*. The absorption peak at 2950–2800 cm⁻^1^ is attributed to C-H stretching vibrations in the fatty acids of the fungal cell membrane [30], with *P. victoriae* displaying a characteristic peak at 2924.30 cm⁻^1^. The amide I band for *P. victoriae* appears in the 1639–1619 cm⁻^1^ range, corresponding mainly to C=O stretching and C-N stretching vibrations. The amide II band appears in the 1554–1543 cm⁻^1^ range due to C-C and C-N stretching and N-H and C-O bending vibrations. The absorption peak in the 1200–900 cm⁻^1^ range is attributed to C-O and C-O-C stretching vibrations of carbohydrates and polysaccharides in the cell wall [31,32]. Peaks in the 1070–1076 cm⁻^1^ range are associated with P=O symmetric stretching of phosphate groups in nucleic acids and phospholipids [33]. Generally, higher concentrations of antimicrobials lead to a stronger antimicrobial effect. Interestingly, increasing the concentrations of SA and YW-5 led to four synergistic combinations of YW-5 and SA (4 mg/mL + 1.1 mg/mL, 4 mg/mL + 0.55 mg/mL, 2 mg/mL + 1.1 mg/mL, and 2 mg/mL + 0.55 mg/mL) exhibiting a stronger damaging effect on *P. victoriae*. In contrast, three additive combinations of YW-5 and SA (8 mg/mL + 2.2 mg/mL, 8 mg/mL + 1.1 mg/mL, and 4 mg/mL + 2.2 mg/mL) resulted in a weaker damaging effect. Moreover, the synergistic combination of YW-5 and SA (4 mg/mL + 1.1 mg/mL) demonstrated a markedly greater damaging effect compared to the three additive combinations, confirming that SA and YW-5 act synergistically against *P. victoriae*. Compared to untreated *P. victoriae*, the characteristic peaks in four regions were reduced for all treated groups, with the YW-5/SA (4.0 mg/mL + 1.1 mg/mL)-treated group showing the most significant reduction. This finding indicates that the cell wall was damaged, membrane fluidity increased, and protein and nucleic acids leaked due to the synergistic antifungal effect. Furthermore, the reduction in intensity observed with SA treatment was greater than that with YW-5 treatment alone, suggesting that SA serves a major function in YW-5/SA treatment, leading to the release of cellular components.

**Figure 5:**
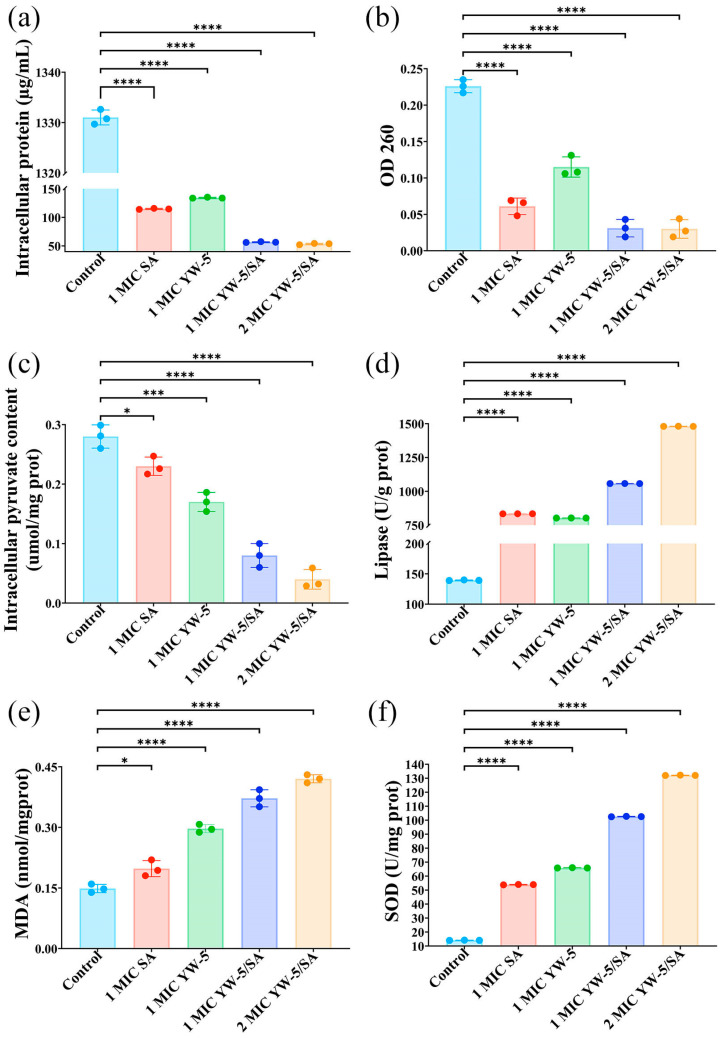
Synergistic effects of YW-5 and SA on the release of cell components and membrane lipid peroxidation. (**a**) Intracellular protein content. (**b**) OD_260_. (**c**) Intracellular pyruvic acid content. (**d**) Lipase activity. (**e**) MDA content. (**f**) SOD activity. * *p* < 0.05, *** *p* < 0.001, and **** *p* < 0.0001.

YW-5/SA markedly (*p* < 0.05) caused leakage of intracellular protein, OD_260_, and pyruvate (Figure 5). Compared to the control group, intracellular protein content decreased by 95.99%, 95.75%, 89.91%, and 91.37% with 2 MIC YW-5/SA, 1 MIC YW-5/SA, SA, and YW-5 treatment, respectively. Additionally, OD_260_ decreased by 86.73%, 86.28%, 73.01%, and 49.12%, respectively, indicating that SA serves a predominant function in the leakage of protein and nucleic acids. Furthermore, YW-5/SA markedly (*p* < 0.05) reduced intracellular pyruvate content, an important intermediate in glucose metabolism. The pyruvate content decreased by 85.71%, 71.43%, 17.86%, and 39.29% with 2 MIC YW-5/SA, 1 MIC YW-5/SA, SA, and YW-5 treatment, respectively. Among them, YW-5 served a major function in reducing intracellular pyruvate content.

### 3.6. The Impact of YW-5 and SA on Membrane Lipid Peroxidation

Lipase is a lipolytic enzyme within the serine hydrolase group, responsible for catalyzing the hydrolysis of ester carboxyl bonds in triacylglycerols. This reaction releases free fatty acids, diacylglycerides, monoglycerides, and glycerol [34]. Compared to untreated *P. victoriae*, lipase activity increased nearly seven-fold with 1 MIC YW-5/SA and approximately ten-fold with 2 MIC YW-5/SA treatments. Among them, SA served a major function, suggesting that YW-5/SA enhances lipase activity and promotes the degradation of membrane phospholipids.

MDA is a by-product of lipid peroxidation whose levels increase in response to reactive oxygen species, leading to damage to phospholipids, enzymes, nucleic acids, and biofilms. MDA levels reflect the extent of lipid peroxidation [19]. Compared to untreated *P. victoriae*, MDA content increased by 33.06%, 99.59%, one-fold, and two-fold with 1 MIC SA, YW-5, YW-5/SA, and 2 MIC YW-5/SA treatment, respectively. This increase indicates that YW-5 contributes more markedly to lipid peroxidation in microbial membranes than SA.

SOD is an endoenzyme responsible for breaking down superoxide radicals in organisms, and excessive SOD activity indicates an imbalance in free radical metabolism, leading to the overproduction of free radicals that can exacerbate membrane lipid peroxidation [35]. All treated groups showed markedly higher SOD activity compared to the control group, indicating that the treatments induced oxidative stress and disrupted the intracellular free radical balance. In particular, the SOD activity in *P. victoriae* treated with 1 MIC YW-5/SA was 90.36% higher than that in the SA-treated group and 55.70% higher than that in the YW-5-treated group, suggesting that YW-5 served a major function in enhancing SOD activity in YW-5/SA treatment.

### 3.7. The Impact of YW-5 and SA on Spore Morphology and Cell Ultrastructure

**Figure 6:**
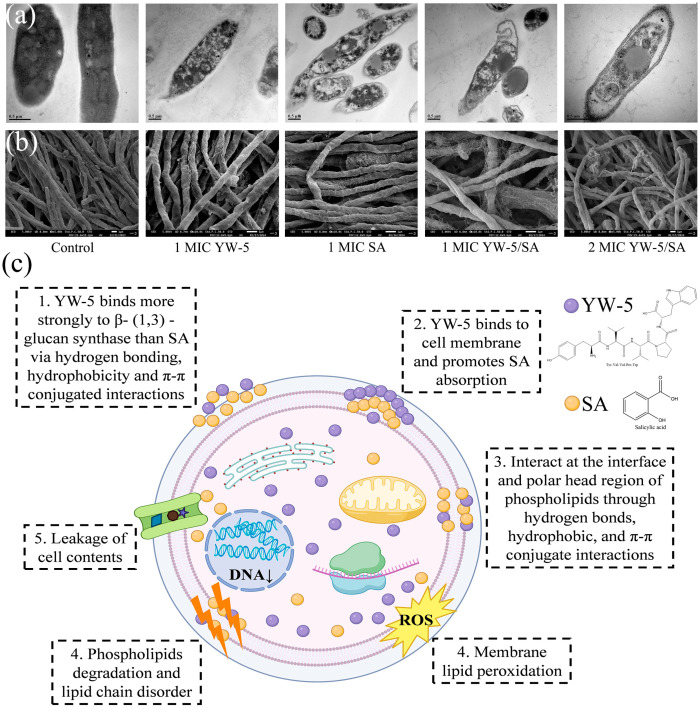
Synergistic effects of YW-5 and SA on the cell ultrastructure (**a**) and spore morphology (**b**) of *P. victoriae.* The proposed mechanism of action for YW-5/SA against *P. victoriae* (**c**).

Untreated *P. victoriae* maintained normal cell morphology, with a clear plasma membrane structure and well-preserved organelles (Figure 6a). After treatment with SA alone, the cell wall became thinner, numerous vacuoles fused in the cytoplasm, and there were notable signs of content leakage around the spores of SA-treated cells. Compared to SA-treated *P. victoriae*, those treated with YW-5 showed slightly less cell wall damage. However, the most severe damage occurred with YW-5/SA treatment, especially at 2 MIC. In this case, the cell wall was notably thinned, the cytoplasmic matrix was reduced, numerous vacuoles fused in the cytoplasm, cell contents leaked, and many organelles were lost.

The SEM images of *P. victoriae* spore morphology are presented in Figure 6b. Spores from untreated *P. victoriae* appeared smooth and full, with no cracks and wrinkles, indicating normal growth. In contrast, YW-5 treatment caused noticeable cracking on the spore surface, while SA treatment resulted in more extensive wrinkling and dissolution. Following treatment with 1 MIC and 2 MIC YW-5/SA, the spores exhibited more pronounced lysis compared to those treated with YW-5 or SA alone, demonstrating a concentration-dependent effect. These observations confirm that YW-5/SA markedly damages spore morphology and cell ultrastructure, with SA serving a leading function, consistent with previous results.

## 4. Conclusions

This study confirms that the combination of YW-5 and SA derived from FW synergistically damages the cell membrane of *P*. *victoriae*. YW-5 and SA interact synergistically at the interface and polar head region of phospholipids. The main secondary structures of YW-5 interacting with the fungal cell membrane and SA are random coil and β-sheets, respectively. YW-5 exhibits a stronger noncovalent interaction with Rho1 GTPase and Trp, Tyr, and Phe in the cell membrane for lipid peroxidation. In contrast, SA mainly disrupts the lipid chain and facilitates the release of cellular components. Figure 6c illustrates the proposed mechanism of action for YW-5/SA against *P*. *victoriae*. Further research will investigate the molecular-level differences between YW-5 and SA in microbial metabolism. The fungistatic combination of SA and YW-5 could provide a reference for reducing the dosage and increasing the antifungal activity of AMPs and phenolic compounds in fruit preservation.

## Figures and Tables

**Table 1:** Secondary structures of YW-5 in solid and liquid states.

Secondary Structure		α-Helix (%)	β-Sheet (%)	β-Turn (%)	Random (%)
FTIR	In its solid state	16.32%	41.18%	18.96%	23.54%
CD	In HCl	3.0%	32.9%	21.8%	42.3%
	In PBS	1.9%	33.6%	18.7%	45.8%
	In TFE	8.0%	38.2%	12.3%	41.6%
	In SA	0.7%	49.0%	26.6%	23.8%

## Data Availability

The original contributions presented in this study are included in the article. Further inquiries can be directed to the corresponding author.

## Abbreviations

The following abbreviations are used in this manuscript:

FW	Fermented walnut
SA	Salicylic acid
YW-5	YVVPW
